# Partnering To Support Needs-Appropriate Housing For Physically Disabled and Older Veterans

**DOI:** 10.1093/geroni/igaf122.1780

**Published:** 2025-12-31

**Authors:** Anushka Sista, Jennifer Palmer, Nathan Boucher

**Affiliations:** Veterans Administration, Bedford, Massachusetts, United States; Veterans Administration, Bedford, Massachusetts, United States; Duke University, Durham, North Carolina, United States

## Abstract

The number of homeless Veterans aged 55 and older increased by 150% from 2010 to 2023, creating a significant housing stability crisis. While the Veterans Health Administration (VHA) provides programs like Housing and Urban Development-Veterans Affairs Supportive Housing (HUD-VASH) to support Veterans transitioning out of homelessness; older Veterans and those living with physical disabilities require specialized assistance to maintain independence. This VHA quality improvement project aimed to identify barriers and facilitators to HUD-VASH teams and community collaboration as they support Veterans living in least restrictive settings and to develop an implementation blueprint to guide optimal collaboration. We conducted qualitative interviews with 18 regional HUD-VASH geriatric specialists across VHA nationally and facilitated user-centered design (UCD) sessions with VHA staff and community agency representatives at two VA medical centers. Our integrated analysis from the interview and UCD datasets used Shepherd et al.’s framework for interagency collaboration, including elements such as Memoranda of Understanding, Shared Goals, Policies and Procedures, Role Descriptions, Information Sharing, and Regular Meetings. Key challenges included communication barriers, administrative complexity, resource limitations, partnership difficulties, and Veteran-specific obstacles. Geographic diversity, unclear role definitions, and lack of centralized information sharing were major barriers to effective collaboration. Many findings applied to multiple framework elements. Our integrated data informed an implementation blueprint for potential implementation by VHA HUD-VASH teams nationwide. This project offers a structured approach to addressing the complex housing needs of aging and physically disabled Veterans through improved community collaboration. Future research should evaluate the effectiveness of the implementation blueprint in practice.

